# Robust sliding-Backstepping mode control of a wind system based on the DFIG generator

**DOI:** 10.1038/s41598-022-15960-7

**Published:** 2022-07-12

**Authors:** Farah Echiheb, Yasmine Ihedrane, Badre Bossoufi, Manale Bouderbala, Saad Motahhir, Mehedi Masud, Sultan Aljahdali, Madiha ElGhamrasni

**Affiliations:** 1 LIMAS Laboratory, Faculty of Sciences Dhar El Mahraz, Sidi Mohammed Ben Abdellah University, Fez, Morocco; 2 Engineering, Systems, and Applications Laboratory, ENSA, SMBA University, Fez, Morocco; 3 Department of Computer Science, College of Computers and Information Technology, Taif University, P. O. Box 11099, Taif, 21944 Saudi Arabia

**Keywords:** Engineering, Electrical and electronic engineering

## Abstract

This paper presents a new contribution in the field of the optimization of the techniques of control of the wind systems and the improvement of the quality of energy produced in the grid. The Sliding Mode control technique gives quite interesting results, but its major drawback lies in the phenomenon of chattering (oscillations), which reduces the system's precision. We propose in this work a solution to cancel this chattering phenomenon by the implication of the adaptive Backstepping technique to control the powers of the double-fed asynchronous generator (DFIG) connected to the electrical network by two converters (network side and side machine) in the nominal part of the sliding mode model. This hybrid technique will correct errors of precision and stability and the performance of the wind system obtained in terms of efficiency, active and reactive power is significant. First, a review of the wind system was presented. Then, an exhaustive explanation of the Backstepping technique based on the Lyapunov stability and optimization method has been reported. Subsequently, a validation on the Matlab & Simulink environment was carried out to test the performance and robustness of the proposed model. The results obtained from this work, either by follow-up or robustness tests, show a significant performance improvement compared to other control techniques.

## Introduction

Faced with a growing demand for energy caused by overpopulation throughout the globe and the Monopoly of countries in the industrial field, and the more or less long-term depletion of fossil fuels, traditional energy sources are less and less abundant, and different alternatives have been considered. For example, following the oil crises, some countries have pursued a nuclear-oriented policy while others have massively used renewable energies with the adoption of wind power^1^.

Three significant families are emerging in the field of renewable energies: energies of mechanical origin (wind), energies of electrical origin (photovoltaic panels) or energy in the form of heat (geothermal, solar thermal, etc.). For centuries, wind energy has been used to provide mechanical work. The best-known example is the windmill. In 1888, Charles F. Brush built a small wind turbine to supply his house with electricity, with battery storage^2^.

The displacement of air masses, which is indirectly due to the Earth's sun, generates wind energy. The warming of some areas of the planet and the cooling of others leads to creating a pressure difference and the movement of air masses in a constant way. The energy recovered depends on the wind speed and the surface facing the wind. This energy is used either directly (milling, pumping) or indirectly (electricity production via a generator).

To start a wind turbine, you need a speed of about 15 km/h^3, 4^. And for safety reasons, the wind turbine must stop when the wind exceeds 90 km/h. In the nacelle, there is an axis called a shaft driven by the rotor of the alternator. The rotation of the shaft provides energy that allows the alternator to produce an alternating electric current. Depending on the strength of the wind, wind turbines rotate more than 80% of the time at varying speeds. In addition, a wind farm with 4 to 6 wind turbines covers the electricity needs of nearly 12,000 people.

Most wind turbines installed in the past were fixed speed^5, 6^. However, these wind turbines have several disadvantages: low energy efficiency and a short lifespan. In addition, this wind turbine technology generates considerable fluctuations in the voltage and power of the network during significant variations in wind speed. Variable-speed wind turbines were then introduced to solve these problems^7, 8^.

The most used wind systems in the industry are based on the DFIG double-fed asynchronous generator, given its advantages of size, cost, efficiency, wide operating range (hyper and hypo synchronous), and its acoustic noise in its four-quadrant operation. As a result, they produce high-quality electrical power compared to fixed-speed wind turbines^9^.

Several research works on the control and control of wind turbines have been carried out^10–13^. Nevertheless, the synthesis of these controls from the linear model of the wind turbine degrades performance, especially when faced with a real wind profile^14–16^. This is due to the strongly non-linear aspect of the wind system. Thus, the impact of disturbances on the system is generally not considered with sufficient precision. Consequently, this type of control does not make it possible to maintain good tracking performance in the presence of external disturbances.

This article deals with the modelling, control and simulation of a wind energy conversion system based on a double-fed asynchronous generator (DFIG); this later is connected directly to the grid by its stator and driven by its rotor by two static converters. The idea is to implement a control system decoupled from DFIG to ensure better power quality and make the system insensitive to disturbances.

Our objective in this work is to:

- Develop non-linear modelling of the wind system based on the DFIG
- Apply the classic model of the sliding mode control technique, and highlight its drawback, which is the phenomenon of chattering (oscillations)
- Develop a very robust hybrid control technique with estimators, which is based on a mixture of the Sliding-Backstepping Mode technique, which significantly improves the performance of the wind system in view of the variation of the wind and also the parameter variation of the machine

This paper is organized as follows: after the introduction, a literature review highlights the problem and then the wind system's dynamic modelling (WECS). Next, the design of the hybrid control in sliding-Backstepping mode is proposed. Finally, the simulation results of the proposed control are presented, analyzed, and then compared to the results of other controls.

## Literature review

This section presents some works in the field of control of wind systems, such as classical sliding mode control, direct torque control, and vector control.

Bossoufi et al.^1^ This paper discussed a non-linear control applied to a WECS-DFIG and developed the Adaptive Backstepping control based on the Lyapunov stability technique to make WECS works in better conditions.

Yang et al.^2^ implemented SMC on DFIG. Unfortunately, the obtained results were inefficient in terms of robustness and set-point follow-up (chattering phenomenon).

Benbouzid et al.^3^ presented a high-order sliding mode control technique to a DFIG-based wind system. The high order has improved the performance of the wind system compared to the classical sliding mode control technique, but the results still show oscillations, and the THD remains high.

Djeriri et al.^4^ presents a work based on artificial intelligence techniques based on neural networks (DTC-RNA). For a DFIG-based wind system, they combined artificial neural networks with DTC of which hysteresis correctors pose the problem of fluctuations on the different output quantities of the DFIG.

In addition to the previously mentioned non-linear control systems, sliding mode control (SMC) has attracted substantial interest because of its organizational efficiency, fast reaction, ease of implementation, and low sensitivity to parameter changes^14, 17^. SMC is a sort of non-linear control that is insensitive to parameter changes. Due to its ease of implementation, order reduction, and tolerance for external disturbances and parametric perturbations, including suitable wind energy extraction, DC link wattage maintenance, and direct wind energy power management, it has attracted considerable interest for WECS control in recent years.

In^18, 19^, sliding mode control (SMC) of the first order is utilized to regulate both the speed and power of the PMSG-based WECS. Typically, the current control creates a real-time voltage reference using pulse-width modulation (PWM). The voltage reference cannot be adequately tracked when the sign function is utilized due to the chattering problem. Using continuous approximation and a saturation function for PMSG-based WECS,^18^ built an SMC with decreased chattering. Unfortunately, applying a saturation function leads to a finite steady-state error. The authors of^11^ provide a way for improving the output power quality using fractional-order sliding mode control (FOSMC); nevertheless, this method requires accurate fractional operator adjustment. The authors of^20^ studied a second-order adaptive SMC approach (SOSMC). This method can successfully account for model error, the inherent nonlinearity of WECS, and random wind. However, due to measurement noise, the use of differentiators demands additional caution.

Meanwhile,^21^ described the I-SMC (integral sliding-model control) approach for achieving high-precision steady-state control. However, the controller's gain must be carefully adjusted to balance volume and noise. In^22^, SOSMC and the Super Twisting (ST) algorithm are merged. Despite the fact that ST can create a quick transient response with low steady-state error, it typically results in high controller gains, which can cause chattering.

Advanced SMC techniques with finite-time convergence have been presented to successfully decrease chattering problems, such as traditional terminal sliding mode control (TSMC) and fast terminal sliding mode control (FTSMC). Both of these solutions rely on non-linear sliding surfaces that incorporate fractional power to enable quick, finite-time convergence during the sliding phase. However, the chattering phenomenon in TSMC and FTSMC is not totally removed, as it is in traditional SMC.

According to these studies, the major problem encountered in most control algorithms was in terms of robustness. Our contribution in this work is to confirm the tracking and regulation performance and then make the wind system insensitive to parametric variations. For that purpose, this study aims to Develop a robust hybrid control technique with estimators, which is based on a mixture of the Sliding-Backstepping Mode technique, which greatly improves the performance of the wind system in view of the variation of the wind and also the parameter variation of the machine.

## Modeling of a wind system based on the DFIG

**Figure 1:**
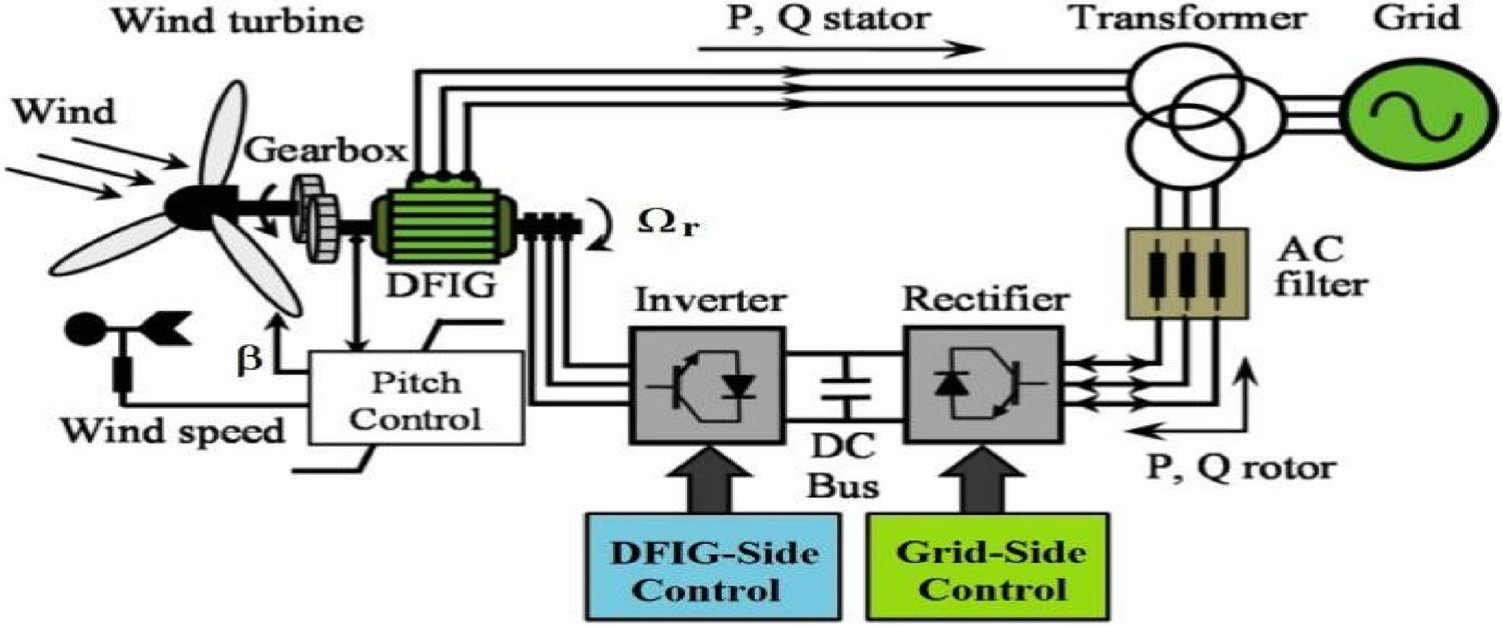
WECS based on the DFIG.

The turbine transforms the kinetic energy of the wind into mechanical energy. It comprises three identical blades fixed to a drive shaft connected to a speed multiplier having a transformation ratio G. This multiplier drives the shaft of the electric generator (Fig. 1)^17^.

**Figure 2:**
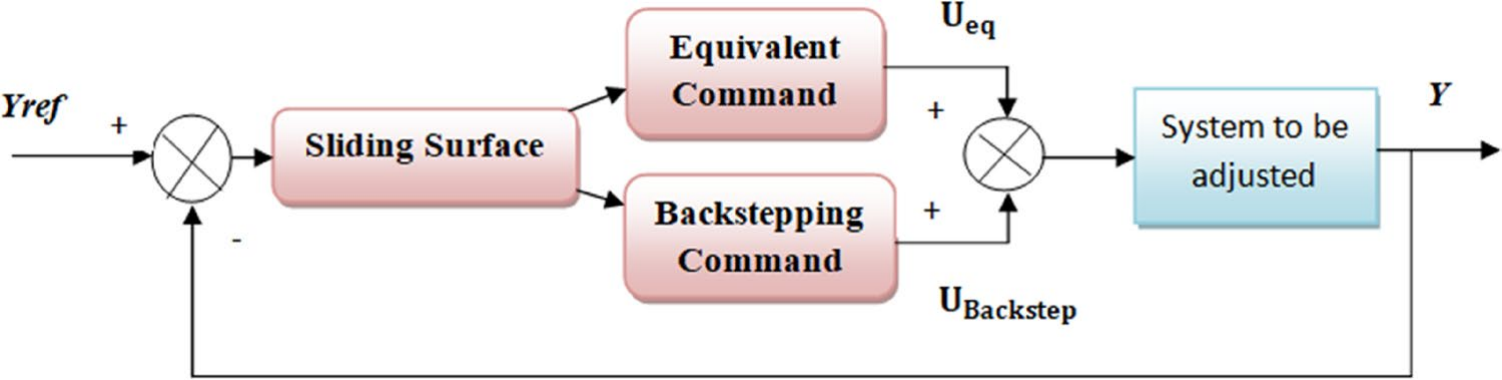
Structure of the Sliding-Backstepping Mode Controller.

**Figure 3:**
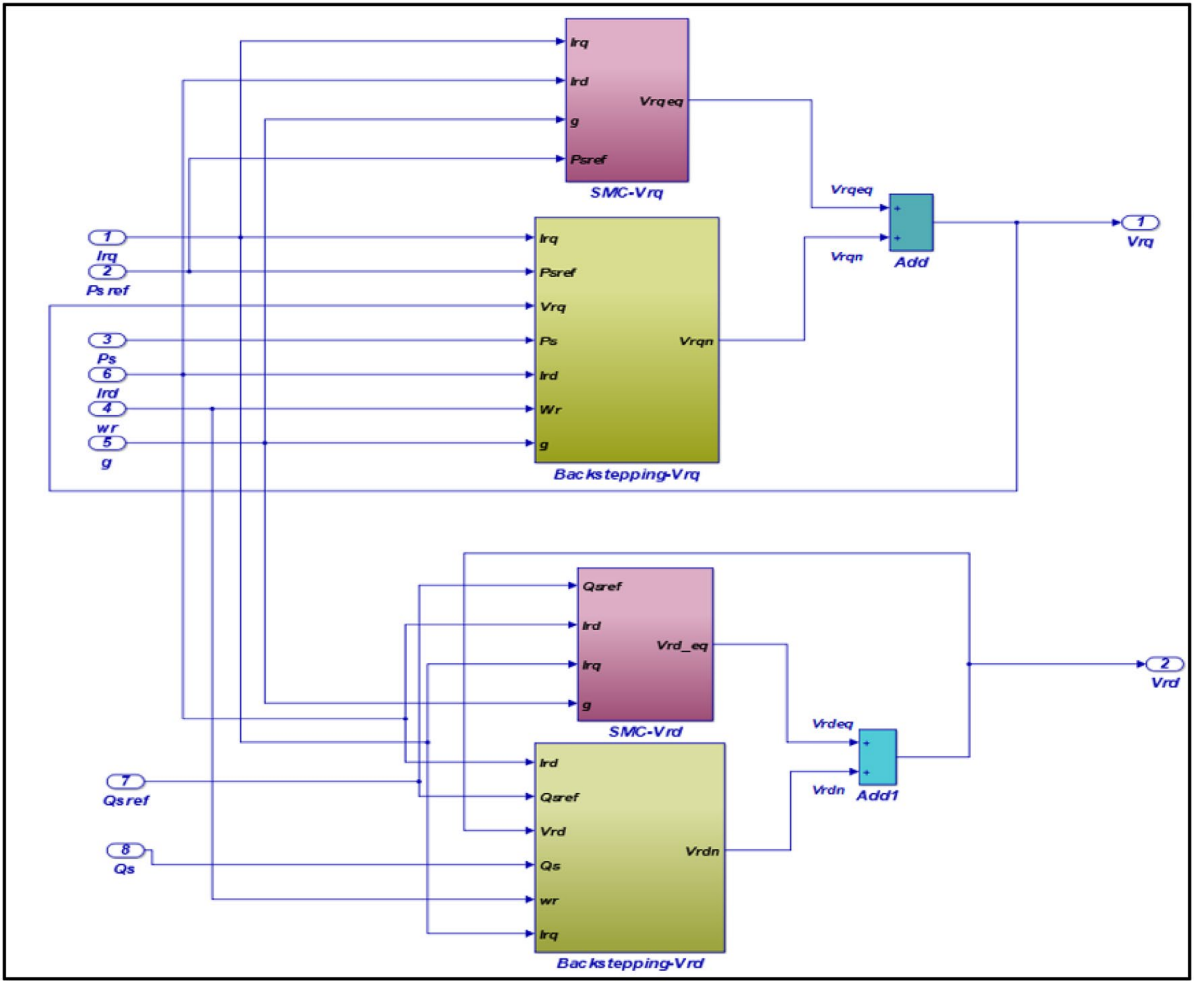
Sliding-Backstepping mode control applied to the RSC.

**Figure 4:**
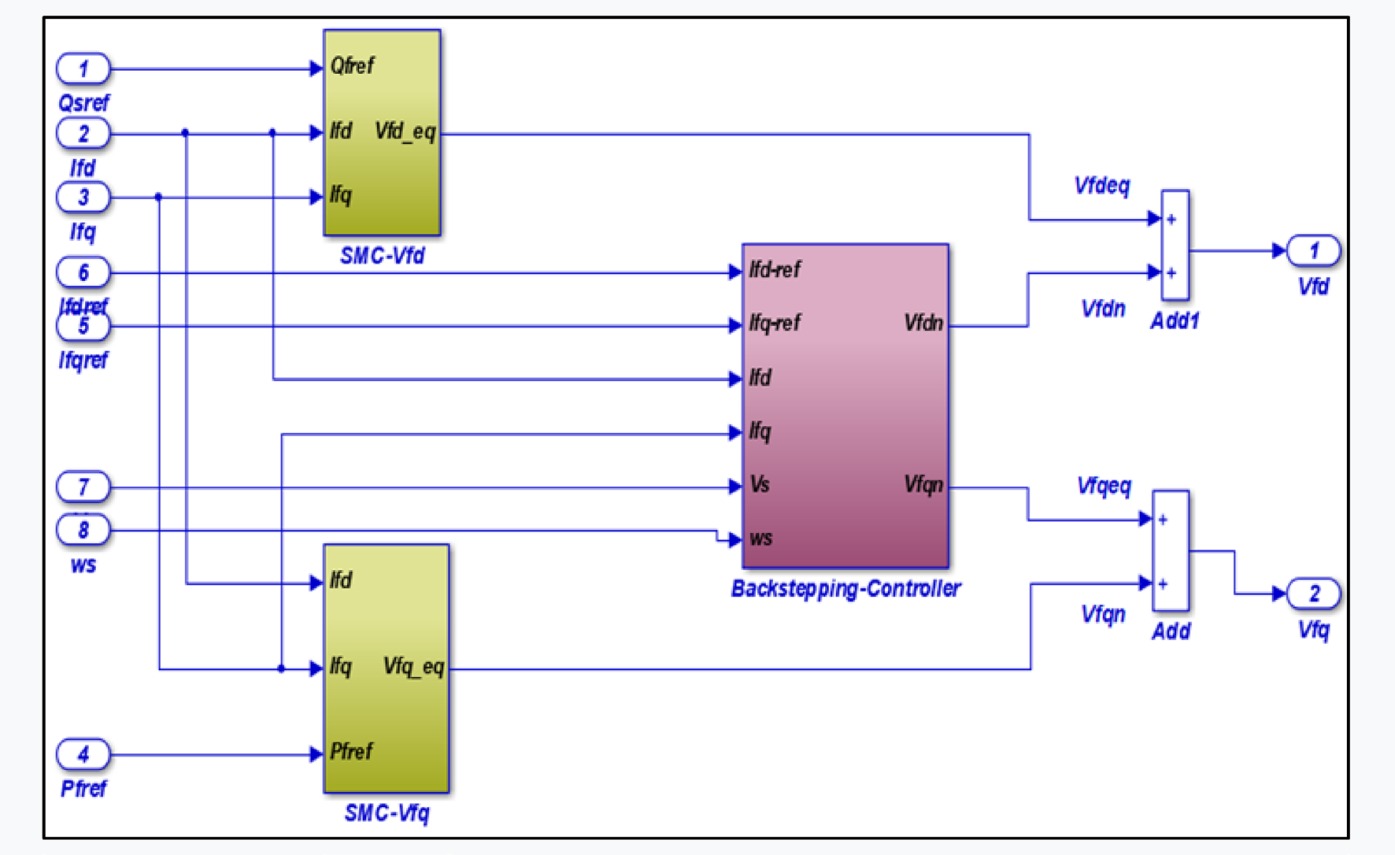
Sliding-Backstepping mode control applied to GSC.

The generator then provides electrical energy at a variable frequency, and it is necessary to add a power electronics interface between it and the network. This interface is conventionally made up of two converters (a rectifier and an inverter) connected via a DC voltage stage. The grid-side inverter is then decoupled from the machine via the DC bus and there is no direct link between the grid frequency and that delivered by the machine. Such a device must however be designed and controlled in such a way as to limit the disturbances that it is liable to generate on the network. Indeed, the voltage delivered is not sinusoidal and may contain undesirable harmonics. In addition, the converters are sized to transmit all the power exchanged between the generator and the grid; they therefore, represent a financial investment and lead to significant losses^18, 19^.

### WECS model

The turbine is modeled by the following system of equations^17, 23^:

1 $$\left\{\begin{array}{c}{\mathrm{P }}_{\mathrm{aero}}=\frac{1}{2}.{\mathrm{C}}_{\mathrm{p}-\mathrm{max}}\left(\uplambda ,\upbeta \right).\rho .\pi .{\mathrm{R}}^{2}.{\mathrm{v}}^{3}\\ \lambda =\frac{\mathrm{R}.\mathrm{\Omega t}}{\mathrm{v}} \\ {\mathrm{C}}_{\mathrm{p}-\mathrm{Max}}=\frac{16}{27}=0.5925\\ \frac{1}{\lambda }=\frac{1}{\uplambda +0.008\upbeta }-\frac{0.035}{{\beta }^{3}+1}\\ {\mathrm{C}}_{\mathrm{p}}\left(\uplambda ,\upbeta \right)={\mathrm{C}}_{1}\left(\frac{{\mathrm{C}}_{2}}{\mathrm{A}}-{\mathrm{C}}_{3}.\upbeta -{\mathrm{C}}_{4}\right){\mathrm{e}}^{\frac{-{\mathrm{C}}_{5}}{\mathrm{A}}}+{\mathrm{C}}_{6}\end{array}\right.$$

where ${\mathrm{C}}_{\mathrm{p}}$: Power coefficient, λ: Relative speed, β: Pitch angle (deg), R: Radius of the turbine, v: Wind speed (m/s), ρ: Air density (1.225 kg/m^3^).

The turbine is connected to the generator shaft through a gearbox whose model is as follows:

2 $$\left\{\begin{array}{c}{\Omega }_{\mathrm{t}}=\frac{{\Omega }_{\mathrm{mec}}}{\mathrm{G}}\\ {\mathrm{C}}_{\mathrm{g}}=\frac{{\mathrm{C}}_{\mathrm{aero}}}{\mathrm{G}}\end{array}\right.$$

The following mechanical equations model the generator shaft:

3 $$\left\{\begin{array}{c}{\mathrm{J}}_{\mathrm{tot}}=\frac{{\mathrm{J}}_{\mathrm{t}}}{{\mathrm{G}}^{2}}+{\mathrm{J}}_{\mathrm{g}}\\ {\mathrm{C}}_{\mathrm{mec}}={\mathrm{J}}_{\mathrm{tot}}.\frac{\mathrm{d}{\Omega }_{\mathrm{mec}}}{\mathrm{dt}}={\mathrm{C}}_{\mathrm{g}}-{\mathrm{C}}_{\mathrm{em}}-{\mathrm{C}}_{\mathrm{f}}\end{array}\right.$$

with: $${\mathrm{C}}_{\mathrm{f}} =\mathrm{f}.{\Omega }_{\mathrm{mec}}$$

### DFIG model

According to the rotating field reference frame of Park, the model of the DFIG is given by the following set of equations^24,20–22,25,26^:

4 $$\left\{\begin{array}{c}{V}_{sd}={R}_{s}.{I}_{sd}+\frac{d{\phi }_{sd}}{dt}-{\omega }_{s}.{\phi }_{sq}\\ {V}_{sq}={R}_{s}.{I}_{sq}+\frac{d{\phi }_{sq}}{dt}+{\omega }_{s}.{\phi }_{sd}\\ {V}_{rd}={R}_{r}.{I}_{rd}+\frac{d{\phi }_{rd}}{dt}-{\omega }_{r}.{\phi }_{rq}\\ {V}_{rq}={R}_{r}.{I}_{rq}+\frac{d{\phi }_{rq}}{dt}+{\omega }_{r}.{\phi }_{rd}\end{array}\right.$$

5 $$\begin{array}{c}{\omega }_{r}={\omega }_{s}-p.\Omega \\ \left\{\begin{array}{c}{\varphi }_{ds}={L}_{s}.{i}_{ds}+M.{i}_{dr}\\ {\varphi }_{qs}={L}_{s}.{i}_{qs}+M.{i}_{qr}\\ {\varphi }_{dr}={L}_{r}.{i}_{dr}+M.{i}_{ds}\\ {\varphi }_{qr}={L}_{r}.{i}_{qr}+M.{i}_{qs}\end{array}\right.\\ {L}_{s}={l}_{s}-{M}_{s},\begin{array}{cc}& {L}_{r}={l}_{r}-{M}_{r}\end{array}\end{array}$$

6 $${C}_{em}=p\left({\phi }_{ds}{i}_{qs}-{\phi }_{qs}{i}_{ds}\right)$$

7 $$\left\{\begin{array}{c}{P}_{s}={v}_{ds}.{i}_{ds}+{v}_{qs}.{i}_{qs}\\ {Q}_{s}={v}_{qs}.{i}_{ds}-{v}_{ds}.{i}_{qs}\\ {P}_{r}={v}_{dr}.{i}_{dr}+{v}_{qr}.{i}_{qr}\\ {Q}_{r}={v}_{qr}.{i}_{dr}-{v}_{dr}.{i}_{qr}\end{array}\right.$$

## Non-linear control of the WECS based on the DFIG

### The principle of SMC”

The sliding mode control consists of two terms^27,28^: a discontinuous control depending on the sign of the sliding surface and an equivalent control characterizing the dynamics of the sliding surface. The system on the sliding surface:

8 $$\mathrm{u}={\mathrm{u}}_{\mathrm{eq}}+{\mathrm{u}}_{\mathrm{n}}$$

${\mathrm{u}}_{\mathrm{eq}}$: The equivalent control vector describes an ideal sliding motion, i.e. without taking into account the uncertainties and disturbances of the system. It is obtained thanks to the following conditions of invariance of the sliding surface:

9 $$\left\{\begin{array}{c}\dot{\begin{array}{c}S\left(\mathrm{x}\right)=0\\ S\left(\mathrm{x}\right)=0\end{array}}\end{array}\right.$$

In the literature, several choices for the discontinuous control ${\mathrm{u}}_{\mathrm{n}}$ are proposed; the simplest one is given by:

10 $${\mathrm{u}}_{\mathrm{n}}=\mathrm{K}.\mathrm{sign}(\mathrm{S}\left(\mathrm{x}\right))$$

With K is the control gain.

The expression of the sliding surface is described as follows:

11 $$\mathrm{S}\left(\mathrm{x}\right)={\left(\frac{\mathrm{d}}{\mathrm{dt}}+\updelta \right)}^{\mathrm{r}-1}*\mathrm{ e}\left(\mathrm{x}\right)$$

$\updelta$ : Positive gain that interprets the bandwidth of the desired control.

$\mathrm{e}\left(\mathrm{x}\right)$ : the difference on the variable to be regulated e(x) = Xref-X.

r: relative degree, the smallest positive integer representing the number of times that must be differentiated to make the control appear.

The convergence condition is defined by the LYAPUNOV function V(x), which makes the surface attractive and invariant^29,30^.

12 $$\mathrm{V}\left(\mathrm{x}\right)=\frac{1}{2} .{\mathrm{S}(\mathrm{x})}^{2}$$

This function is obviously positive definite. The necessary and sufficient condition for the slip variable S(x, t) to tend to zero is that its derivative be negative definite:

13 $$\dot{\mathrm{V}(\mathrm{x})}=\mathrm{S}\left(\mathrm{x}\right)\dot{*\mathrm{S}\left(\mathrm{x}\right)<0}$$

The main drawback of this type of control is the phenomenon known as “CHATTERING”^31^. In addition, due to the discontinuous nature of the "SIGN" function, high-frequency oscillations around the equilibrium point appear in a steady-state^32^.

### Hybrid sliding-Backstepping mode control

To remedy the chattering problem of sliding mode control, we are interested in the new control technique proposed in this article which improves performance and reduces this phenomenon. This technique is called the hybrid Sliding-Backstepping Mode control of DFIG.

The principle of the hybrid control consists in modifying the controller by sliding mode by replacing the stabilizing control ${\mathrm{u}}_{\mathrm{n}}$ of the sliding mode regulator by the Backstepping controller to solve the CHATTERING problem. The combination of the two parts thus makes it possible to ensure the stability and the robustness of the studied system.

The structure of the Sliding-Backstepping Mode controller is shown in Fig. 2.

This technique is based on decomposing the entire control system, which is usually multivariate and high order into a cascade of first-order control subsystems. Next, a virtual control law is calculated for each subsystem. The latter is considered as a reference for the next subsystem until the control law for the complete system is obtained^33, 34^.

#### Applying the Backstepping control to the RSC”

**Step 1**: Calculation of reference rotor currents

We define the error “${\mathrm{e}}_{1}$” and “${\mathrm{e}}_{2}$” as follows:

14 $$\left\{\begin{array}{c} \\ {\mathrm{e}}_{1}={\mathrm{P}}_{\mathrm{sref}}-{\mathrm{P}}_{\mathrm{s}}\\ {\mathrm{e}}_{2}={\mathrm{Q}}_{\mathrm{sref}}-{\mathrm{Q}}_{\mathrm{s}}\end{array}\right.$$

15 $$\left\{\begin{array}{c}{\dot{\mathrm{e}}}_{1}={\dot{\mathrm{P}}}_{\mathrm{sref}}-{\dot{\mathrm{P}}}_{\mathrm{s}}\\ {\dot{\mathrm{e}}}_{2}={\dot{\mathrm{Q}}}_{\mathrm{sref}}-{\dot{\mathrm{Q}}}_{\mathrm{s}}\end{array}\right.$$

with:

16 $$\left\{\begin{array}{c}{\dot{\mathrm{P}}}_{\mathrm{s}}=-\frac{{\mathrm{v}}_{\mathrm{s}}.\mathrm{M}}{{\mathrm{L}}_{\mathrm{s}}}{\dot{\mathrm{I}}}_{\mathrm{rq}}\\ {\dot{\mathrm{Q}}}_{\mathrm{s}}=\frac{{\mathrm{v}}_{\mathrm{s}}^{2}}{{\upomega }_{\mathrm{s}}.{\mathrm{L}}_{\mathrm{s}}}-\frac{{\mathrm{v}}_{\mathrm{s}}.\mathrm{M}}{{\mathrm{L}}_{\mathrm{s}}}{\dot{\mathrm{I}}}_{\mathrm{rd}}\\ \begin{array}{c}{\dot{\mathrm{I}}}_{\mathrm{rd}}=\frac{{\mathrm{V}}_{\mathrm{rd}}}{{\mathrm{L}}_{\mathrm{r}}.\upsigma }-\frac{{\mathrm{R}}_{\mathrm{r}}}{{\mathrm{L}}_{\mathrm{r}}.\upsigma }.{\mathrm{I}}_{\mathrm{rd}}+{\upomega }_{\mathrm{r}}.{\mathrm{I}}_{\mathrm{rq}}\\ \begin{array}{c}{\dot{\mathrm{I}}}_{\mathrm{rq}}=\frac{{\mathrm{V}}_{\mathrm{rq}}}{{\mathrm{L}}_{\mathrm{r}}.\upsigma }-\frac{{\mathrm{R}}_{\mathrm{r}}}{{\mathrm{L}}_{\mathrm{r}}.\upsigma }.{\mathrm{I}}_{\mathrm{rq}}-{\upomega }_{\mathrm{r}}.{\mathrm{I}}_{\mathrm{rd}}-{\upomega }_{\mathrm{r}}.\frac{\mathrm{M}.{\mathrm{V}}_{\mathrm{s}}}{{\mathrm{L}}_{\mathrm{r}}.{\mathrm{L}}_{\mathrm{s}}.\upsigma .{\upomega }_{\mathrm{s}}}\end{array}\end{array}\end{array}\right.$$

17 $$\left\{\begin{array}{c}{\dot{\mathrm{e}}}_{1}={\dot{\mathrm{P}}}_{\mathrm{sref}}+\frac{{\mathrm{V}}_{\mathrm{s}}.\mathrm{M}}{{\mathrm{L}}_{\mathrm{s}}}(\frac{{\mathrm{V}}_{\mathrm{rq}}}{{\mathrm{L}}_{\mathrm{r}}.\upsigma }-\frac{{\mathrm{R}}_{\mathrm{r}}}{{\mathrm{L}}_{\mathrm{r}}.\upsigma }.{\mathrm{I}}_{\mathrm{rq}}-{\upomega }_{\mathrm{r}}.{\mathrm{I}}_{\mathrm{rd}}-{\upomega }_{\mathrm{r}}.\frac{\mathrm{M}.{\mathrm{V}}_{\mathrm{s}}}{{\mathrm{L}}_{\mathrm{r}}.{\mathrm{L}}_{\mathrm{s}}.\upsigma .{\upomega }_{\mathrm{s}}})\\ {\dot{\mathrm{e}}}_{2}={\dot{\mathrm{Q}}}_{\mathrm{sref}}+\frac{{\mathrm{V}}_{\mathrm{s}}.\mathrm{M}}{{\mathrm{L}}_{\mathrm{s}}}(\frac{{\mathrm{V}}_{\mathrm{rd}}}{{\mathrm{L}}_{\mathrm{r}}.\upsigma }-\frac{{\mathrm{R}}_{\mathrm{r}}}{{\mathrm{L}}_{\mathrm{r}}.\upsigma }.{\mathrm{I}}_{\mathrm{rd}}+{\upomega }_{\mathrm{r}}.{\mathrm{I}}_{\mathrm{rq}})\end{array}\right.$$

$$\upsigma =1-\frac{{M}^{2}}{{L}_{s}.{L}_{r}}$$

The Lyapunov function associated with the errors of the active and reactive power of the stator is given by the following equation:

18 $${\mathrm{V}}_{1}=\frac{1}{2}{\mathrm{e}}_{1}^{2}+\frac{1}{2}{\mathrm{e}}_{2}^{2}$$

Its derivative is expressed by:

19 $${\dot{\mathrm{V}}}_{1} ={\mathrm{e}}_{1}{\dot{\mathrm{e}}}_{1}+{\mathrm{e}}_{2}{\dot{\mathrm{e}}}_{2}{ =\mathrm{e}}_{1}\left[{\dot{\mathrm{P}}}_{\mathrm{sref}}+\frac{{\mathrm{V}}_{\mathrm{s}}.\mathrm{M}}{{{\mathrm{L}}_{\mathrm{r}}.\upsigma .\mathrm{L}}_{\mathrm{s}}}\left({\mathrm{V}}_{\mathrm{rq}}-{\mathrm{R}}_{\mathrm{r}}.{\mathrm{I}}_{\mathrm{rq}}-{{\mathrm{L}}_{\mathrm{r}}.\upsigma .\upomega }_{\mathrm{r}}.{\mathrm{I}}_{\mathrm{rd}}-\mathrm{g}.\frac{\mathrm{M}.{\mathrm{V}}_{\mathrm{s}}}{{\mathrm{L}}_{\mathrm{s}}}\right)\right]+ {\mathrm{e}}_{2}\left[{\dot{\mathrm{Q}}}_{\mathrm{sref}}+\frac{{\mathrm{V}}_{\mathrm{s}}.\mathrm{M}}{{\mathrm{L}}_{\mathrm{r}}.\upsigma .{\mathrm{L}}_{\mathrm{s}}}\left({\mathrm{V}}_{\mathrm{rd}}-{\mathrm{R}}_{\mathrm{r}}.{\mathrm{I}}_{\mathrm{rd}}+{{\mathrm{L}}_{\mathrm{r}}.\upsigma .\upomega }_{\mathrm{r}}.{\mathrm{I}}_{\mathrm{rq}}\right)\right]$$

To ensure the stability of the subsystem, according to Lyapunov ${\dot{\mathrm{V}}}_{1}$ must be negative. For this, we choose it in the form^35–37^:

20 $${\dot{\mathrm{V}}}_{1}=-{\mathrm{k}}_{1}{\mathrm{e}}_{1}^{2}-{\mathrm{k}}_{2}{\mathrm{e}}_{2}^{2}\le 0$$

Performing the equality between Eqs. (19) and (20), we obtain:

21 $${\mathrm{e}}_{1}\left[{\dot{\mathrm{P}}}_{\mathrm{sref}}+\frac{{\mathrm{V}}_{\mathrm{s}}.\mathrm{M}}{{{\mathrm{L}}_{\mathrm{r}}.\upsigma .\mathrm{L}}_{\mathrm{s}}}\left({\mathrm{V}}_{\mathrm{rq}}-{\mathrm{R}}_{\mathrm{r}}.{\mathrm{I}}_{\mathrm{rq}}-{{\mathrm{L}}_{\mathrm{r}}.\upsigma .\upomega }_{\mathrm{r}}.{\mathrm{I}}_{\mathrm{rd}}-\mathrm{g}.\frac{\mathrm{M}.{\mathrm{V}}_{\mathrm{s}}}{{\mathrm{L}}_{\mathrm{s}}}\right)\right]+{\mathrm{e}}_{2}\left[{\dot{\mathrm{Q}}}_{\mathrm{sref}}+\frac{{\mathrm{V}}_{\mathrm{s}}.\mathrm{M}}{{\mathrm{L}}_{\mathrm{r}}.\upsigma .{\mathrm{L}}_{\mathrm{s}}}\left({\mathrm{V}}_{\mathrm{rd}}-{\mathrm{R}}_{\mathrm{r}}.{\mathrm{I}}_{\mathrm{rd}}+{{\mathrm{L}}_{\mathrm{r}}.\upsigma .\upomega }_{\mathrm{r}}.{\mathrm{I}}_{\mathrm{rq}}\right)\right]=-{\mathrm{k}}_{1}{\mathrm{e}}_{1}^{2}-{\mathrm{k}}_{2}{\mathrm{e}}_{2}^{2}$$

Which give:

22 $$\begin{array}{c}{\mathrm{e}}_{1}\left[{\dot{\mathrm{P}}}_{\mathrm{sref}}+\frac{{\mathrm{V}}_{\mathrm{s}}.\mathrm{M}}{{{\mathrm{L}}_{\mathrm{r}}.\upsigma .\mathrm{L}}_{\mathrm{s}}}\left({\mathrm{V}}_{\mathrm{rq}}-{\mathrm{R}}_{\mathrm{r}}.{\mathrm{I}}_{\mathrm{rq}}-{{\mathrm{L}}_{\mathrm{r}}.\upsigma .\upomega }_{\mathrm{r}}.{\mathrm{I}}_{\mathrm{rd}}-\mathrm{g}.\frac{\mathrm{M}.{\mathrm{V}}_{\mathrm{s}}}{{\mathrm{L}}_{\mathrm{s}}}\right)\right]=-{\mathrm{k}}_{1}{\mathrm{e}}_{1}^{2}\\ {\mathrm{e}}_{2}\left[{\dot{\mathrm{Q}}}_{\mathrm{sref}}+\frac{{\mathrm{V}}_{\mathrm{s}}.\mathrm{M}}{{\mathrm{L}}_{\mathrm{r}}.\upsigma .{\mathrm{L}}_{\mathrm{s}}}\left({\mathrm{V}}_{\mathrm{rd}}-{\mathrm{R}}_{\mathrm{r}}.{\mathrm{I}}_{\mathrm{rd}}+{{\mathrm{L}}_{\mathrm{r}}.\upsigma .\upomega }_{\mathrm{r}}.{\mathrm{I}}_{\mathrm{rq}}\right)\right]=-{\mathrm{k}}_{2}{\mathrm{e}}_{2}^{2}\end{array}$$

The expression of the virtual control ${I}_{rq}$ ${I}_{rd}$ and I_rd is defined by:

23 $$\begin{array}{c}{\mathrm{I}}_{\mathrm{rqref}}=\left[\frac{{{\mathrm{L}}_{\mathrm{r}}.\upsigma .\mathrm{L}}_{\mathrm{s}}}{{{\mathrm{R}}_{\mathrm{r}}.\mathrm{V}}_{\mathrm{s}}.\mathrm{M}}\left({\dot{\mathrm{P}}}_{\mathrm{sref}}+{\mathrm{k}}_{1}{\mathrm{e}}_{1}+\frac{{\mathrm{V}}_{\mathrm{s}}.\mathrm{M}}{{{\mathrm{L}}_{\mathrm{r}}.\upsigma .\mathrm{L}}_{\mathrm{s}}}.{(\mathrm{V}}_{\mathrm{rq}}-{{\mathrm{L}}_{\mathrm{r}}.\upsigma .\upomega }_{\mathrm{r}}.{\mathrm{I}}_{\mathrm{rd}}-\mathrm{g}.\frac{\mathrm{M}.{\mathrm{V}}_{\mathrm{s}}}{{\mathrm{L}}_{\mathrm{s}}}\right)\right]\\ {\mathrm{I}}_{\mathrm{rdref}}=\frac{{{\mathrm{L}}_{\mathrm{r}}.\upsigma .\mathrm{L}}_{\mathrm{s}}}{{{\mathrm{R}}_{\mathrm{r}}.\mathrm{V}}_{\mathrm{s}}.\mathrm{M}}\left[{\dot{\mathrm{Q}}}_{\mathrm{sref}}+{\mathrm{k}}_{2}{\mathrm{e}}_{2}+\frac{{\mathrm{V}}_{\mathrm{s}}.\mathrm{M}}{{\mathrm{L}}_{\mathrm{r}}.\upsigma .{\mathrm{L}}_{\mathrm{s}}}\left({\mathrm{V}}_{\mathrm{rd}}+{{\mathrm{L}}_{\mathrm{r}}.\upsigma .\upomega }_{\mathrm{r}}.{\mathrm{I}}_{\mathrm{rq}}\right)\right]\end{array}$$

This will be the desired system reference that follows.

**Step 2**: Calculation of rotor voltages

We will deduce the true control law ${\mathrm{V}}_{\mathrm{rqn}}$ and ${\mathrm{V}}_{\mathrm{rdn}}$ which makes it possible to achieve the design objectives for the overall system.

The rotor current errors are defined by^38, 39^:

24 $$\left\{\begin{array}{c}{\mathrm{e}}_{3}={\mathrm{I}}_{\mathrm{rqref}}-{\mathrm{I}}_{\mathrm{rq}}\\ {\mathrm{e}}_{4}={\mathrm{I}}_{\mathrm{rdref}}-{\mathrm{I}}_{\mathrm{rd}}\end{array}\right..$$

Their derivatives are given by:

25 $$\left\{\begin{array}{c}{\dot{\mathrm{e}}}_{3}={\dot{\mathrm{I}}}_{\mathrm{rqref}}-{\dot{\mathrm{I}}}_{\mathrm{rq}}\\ {\dot{\mathrm{e}}}_{4}={\dot{\mathrm{I}}}_{\mathrm{rdref}}-{\dot{\mathrm{I}}}_{\mathrm{rd}}\end{array}\right..$$

So:

26 $$\begin{array}{c}{\dot{\mathrm{e}}}_{3}={\dot{\mathrm{I}}}_{\mathrm{rqref}}-\frac{1}{{\mathrm{L}}_{\mathrm{r}}.\upsigma }({\mathrm{V}}_{\mathrm{rqn}}-{\mathrm{R}}_{\mathrm{r}}.{\mathrm{I}}_{\mathrm{rq}}-{{\mathrm{L}}_{\mathrm{r}}.\upsigma .\upomega }_{\mathrm{r}}.{\mathrm{I}}_{\mathrm{rd}}-g.\frac{\mathrm{M}.{\mathrm{V}}_{\mathrm{s}}}{{\mathrm{L}}_{\mathrm{s}}}\\ {\dot{\mathrm{e}}}_{4}={\dot{\mathrm{I}}}_{\mathrm{rdref}}-\frac{1}{{\mathrm{L}}_{\mathrm{r}}.\upsigma }({\mathrm{V}}_{\mathrm{rdn}}-{\mathrm{R}}_{\mathrm{r}}.{\mathrm{I}}_{\mathrm{rd}}+{\mathrm{L}}_{\mathrm{r}}.\sigma .{\upomega }_{\mathrm{r}}.{\mathrm{I}}_{\mathrm{rq}}\end{array}.$$

The extended Lyapunov function becomes as follows:

27 $${\mathrm{ V}}_{2}=\frac{1}{2}{\mathrm{e}}_{1}^{2}+\frac{1}{2}{\mathrm{e}}_{2}^{2}+\frac{1}{2}{\mathrm{e}}_{3}^{2}+\frac{1}{2}{\mathrm{e}}_{4}^{2}.$$

Its derivative is given by:

28 $${\dot{\mathrm{V}}}_{2}={\mathrm{e}}_{1}{\dot{\mathrm{e}}}_{1}+{\mathrm{e}}_{2}{\dot{\mathrm{e}}}_{2}+{\mathrm{e}}_{3}{\dot{\mathrm{e}}}_{3}+{\mathrm{e}}_{4}{\dot{\mathrm{e}}}_{4}$$

Which give:

29 $${\dot{\mathrm{V}}}_{2}={\dot{\mathrm{V}}}_{1}+{\mathrm{e}}_{3}{\dot{\mathrm{e}}}_{3}+{\mathrm{e}}_{4}{\dot{\mathrm{e}}}_{4}={\dot{\mathrm{V}}}_{1}+{\mathrm{e}}_{3}\left[{\dot{\mathrm{I}}}_{\mathrm{rqref}}-\frac{1}{{\mathrm{L}}_{\mathrm{r}}.\upsigma }\left({\mathrm{V}}_{\mathrm{rqn}}-{\mathrm{R}}_{\mathrm{r}}.{\mathrm{I}}_{\mathrm{rq}}-{{\mathrm{L}}_{\mathrm{r}}.\upsigma .\upomega }_{\mathrm{r}}.{\mathrm{I}}_{\mathrm{rd}}-\mathrm{g}.\frac{\mathrm{M}.{\mathrm{V}}_{\mathrm{s}}}{{\mathrm{L}}_{\mathrm{s}}}\right)\right]+{ \mathrm{ e}}_{4}\left[{\dot{\mathrm{I}}}_{\mathrm{rdref}}-\frac{1}{{\mathrm{L}}_{\mathrm{r}}.\upsigma }\left({\mathrm{V}}_{\mathrm{rdn}}-{\mathrm{R}}_{\mathrm{r}}.{\mathrm{I}}_{\mathrm{rd}}+{\mathrm{L}}_{\mathrm{r}}.\upsigma .{\upomega }_{\mathrm{r}}.{\mathrm{I}}_{\mathrm{rq}}\right)\right]$$

${\dot{V}}_{2}$ must be negative for the system to be stable. For this, we choose ${\dot{V}}_{2}$ in the form:

30 $${\dot{\mathrm{V}}}_{2}=-{\mathrm{k}}_{1}{\mathrm{e}}_{1}^{2}-{\mathrm{k}}_{2}{\mathrm{e}}_{2}^{2}-{\mathrm{k}}_{3}{\mathrm{e}}_{3}^{2}-{\mathrm{k}}_{4}{\mathrm{e}}_{4}^{2}={\dot{\mathrm{V}}}_{1}-{\mathrm{k}}_{3}{\mathrm{e}}_{3}^{2}-{\mathrm{k}}_{4}{\mathrm{e}}_{4}^{2}\le 0$$

By making the equality between (29) and (30), we obtain:

31 $${\dot{\mathrm{V}}}_{1}-{\mathrm{k}}_{3}{\mathrm{e}}_{3}^{2}-{\mathrm{k}}_{4}{\mathrm{e}}_{4}^{2}={\dot{\mathrm{V}}}_{1}+{\mathrm{e}}_{3}\left[{\dot{\mathrm{I}}}_{\mathrm{rqref}}-\frac{1}{{\mathrm{L}}_{\mathrm{r}}.\upsigma }\left({\mathrm{V}}_{\mathrm{rqn}}-{\mathrm{R}}_{\mathrm{r}}.{\mathrm{I}}_{\mathrm{rq}}-{{\mathrm{L}}_{\mathrm{r}}.\upsigma .\upomega }_{\mathrm{r}}.{\mathrm{I}}_{\mathrm{rd}}-\mathrm{g}.\frac{\mathrm{M}.{\mathrm{V}}_{\mathrm{s}}}{{\mathrm{L}}_{\mathrm{s}}}\right)\right]+{\mathrm{e}}_{4}\left[{\dot{\mathrm{I}}}_{\mathrm{rdref}}- \frac{1}{{\mathrm{L}}_{\mathrm{r}}.\upsigma }\left({\mathrm{V}}_{\mathrm{rdn}}-{\mathrm{R}}_{\mathrm{r}}.{\mathrm{I}}_{\mathrm{rd}}+{\mathrm{L}}_{\mathrm{r}}.\upsigma .{\upomega }_{\mathrm{r}}.{\mathrm{I}}_{\mathrm{rq}}\right)\right]$$

So:

32 $$\begin{array}{c}{\mathrm{e}}_{3}\left[{\dot{\mathrm{I}}}_{\mathrm{rqref}}-\frac{1}{{\mathrm{L}}_{\mathrm{r}}.\upsigma }\left({\mathrm{V}}_{\mathrm{rqn}}-{\mathrm{R}}_{\mathrm{r}}.{\mathrm{I}}_{\mathrm{rq}}-{{\mathrm{L}}_{\mathrm{r}}.\upsigma .\upomega }_{\mathrm{r}}.{\mathrm{I}}_{\mathrm{rd}}-\mathrm{g}.\frac{\mathrm{M}.{\mathrm{V}}_{\mathrm{s}}}{{\mathrm{L}}_{\mathrm{s}}}\right)\right]=-{\mathrm{k}}_{3}{\mathrm{e}}_{3}^{2}\\ {\mathrm{e}}_{4}\left[{\dot{\mathrm{I}}}_{\mathrm{rdref}}-\frac{1}{{\mathrm{L}}_{\mathrm{r}}.\upsigma }\left({\mathrm{V}}_{\mathrm{rdn}}-{\mathrm{R}}_{\mathrm{r}}.{\mathrm{I}}_{\mathrm{rd}}+{\mathrm{L}}_{\mathrm{r}}.\upsigma .{\upomega }_{\mathrm{r}}.{\mathrm{I}}_{\mathrm{rq}}\right)\right]=-{\mathrm{k}}_{4}{\mathrm{e}}_{4}^{2}\end{array}$$

Which gives the expression of the global real control ${V}_{rqn}$ and ${V}_{rdn}$ defined by:

33 $$\left\{\begin{array}{c}{\mathrm{V}}_{\mathrm{rdn}}={\mathrm{L}}_{\mathrm{r}}.\sigma \left[{\mathrm{k}}_{4}{\mathrm{e}}_{4}+{\dot{\mathrm{I}}}_{\mathrm{rdref}}+\frac{1}{{\mathrm{L}}_{\mathrm{r}}.\upsigma }\left({\mathrm{R}}_{\mathrm{r}}.{\mathrm{I}}_{\mathrm{rd}}-{\mathrm{L}}_{\mathrm{r}}.\upsigma .{\upomega }_{\mathrm{r}}.{\mathrm{I}}_{\mathrm{rq}}\right)\right]\\ {\mathrm{V}}_{\mathrm{rqn}}={\mathrm{L}}_{\mathrm{r}}.\sigma [{\mathrm{k}}_{3}{\mathrm{e}}_{3}+{\dot{\mathrm{I}}}_{\mathrm{rqref}}+\frac{1}{{\mathrm{L}}_{\mathrm{r}}.\upsigma }\left({\mathrm{R}}_{\mathrm{r}}.{\mathrm{I}}_{\mathrm{rq}}+{{\mathrm{L}}_{\mathrm{r}}.\upsigma .\upomega }_{\mathrm{r}}.{\mathrm{I}}_{\mathrm{rd}}+\mathrm{g}.\frac{\mathrm{M}.{\mathrm{V}}_{\mathrm{s}}}{{\mathrm{L}}_{\mathrm{s}}}\right)]\end{array}\right.$$

With: ${\mathrm{k}}_{3}$, ${\mathrm{k}}_{4}$ are positive constants.

#### Applying the Backstepping control to the GSC

**Step 1**: Calculation of filter currents Ifd and Ifq

The active and reactive power errors of the filter are given by:

34 $$\left\{\begin{array}{c}{\mathrm{e}}_{5}={\mathrm{P}}_{\mathrm{fref}}-{\mathrm{P}}_{\mathrm{f}}\\ {\mathrm{e}}_{6}={\mathrm{Q}}_{\mathrm{fref}}-{\mathrm{Q}}_{\mathrm{f}}\end{array}\right.$$

The derivatives of the errors are as follows:

35 $$\left\{\begin{array}{c}{\dot{\mathrm{e}}}_{5}={\dot{\mathrm{P}}}_{\mathrm{fref}}-{\dot{\mathrm{P}}}_{\mathrm{f}}\\ {\dot{\mathrm{e}}}_{6}={\dot{\mathrm{Q}}}_{\mathrm{qfref}}-{\dot{\mathrm{Q}}}_{\mathrm{qf}}\end{array}\right..$$

With :

36 $$\left\{\begin{array}{c}{\mathrm{P}}_{\mathrm{f}}={\mathrm{v}}_{\mathrm{s}}.{\mathrm{I}}_{\mathrm{qf}}\\ {\mathrm{Q}}_{\mathrm{f}}={-\mathrm{v}}_{\mathrm{s}}.{\mathrm{I}}_{\mathrm{df}}\\ {\dot{\mathrm{I}}}_{\mathrm{qf}}=-\frac{{\mathrm{v}}_{\mathrm{qfn}}}{{\mathrm{L}}_{\mathrm{f}}}-\frac{{\mathrm{R}}_{\mathrm{f}}}{{\mathrm{L}}_{\mathrm{f}}}{\mathrm{I}}_{\mathrm{qf}}-{\upomega }_{\mathrm{s}}{\mathrm{I}}_{\mathrm{df}}+\frac{{\mathrm{v}}_{\mathrm{s}}}{{\mathrm{L}}_{\mathrm{f}}}\\ {\dot{\mathrm{I}}}_{\mathrm{df}}=-\frac{{\mathrm{v}}_{\mathrm{dfn}}}{{\mathrm{L}}_{\mathrm{f}}}-\frac{{\mathrm{R}}_{\mathrm{f}}}{{\mathrm{L}}_{\mathrm{f}}}{\mathrm{I}}_{\mathrm{df}}+{\upomega }_{\mathrm{s}}{\mathrm{I}}_{\mathrm{qf}}\end{array}\right.$$

Substituting (36) into (35), we get the following equation:

37 $$\left\{\begin{array}{c}{\dot{\mathrm{e}}}_{5}={\dot{\mathrm{P}}}_{\mathrm{fref}}-{\mathrm{v}}_{\mathrm{s}}(-\frac{{\mathrm{v}}_{\mathrm{qfn}}}{{\mathrm{L}}_{\mathrm{f}}}-\frac{{\mathrm{R}}_{\mathrm{f}}}{{\mathrm{L}}_{\mathrm{f}}}{\mathrm{I}}_{\mathrm{qf}}-{\upomega }_{\mathrm{s}}{\mathrm{I}}_{\mathrm{df}}+\frac{{\mathrm{v}}_{\mathrm{s}}}{{\mathrm{L}}_{\mathrm{f}}})\\ {\dot{\mathrm{e}}}_{6}={\dot{\mathrm{Q}}}_{\mathrm{fref}}+{\mathrm{v}}_{\mathrm{s}}.(-\frac{{\mathrm{v}}_{\mathrm{dfn}}}{{\mathrm{L}}_{\mathrm{f}}}-\frac{{\mathrm{R}}_{\mathrm{f}}}{{\mathrm{L}}_{\mathrm{f}}}{\mathrm{I}}_{\mathrm{df}}+{\upomega }_{\mathrm{s}}{\mathrm{I}}_{\mathrm{qf}})\end{array}\right.$$

The Lyapunov function associated with the errors of the active and reactive power of the filter is given by the following equation^40^:

38 $${\mathrm{V}}_{3}=\frac{1}{2}{\mathrm{e}}_{5}^{2}+\frac{1}{2}{\mathrm{e}}_{6}^{2}.$$

Its derivative is given by:

39 $${\dot{\mathrm{V}}}_{3}={\mathrm{e}}_{5}{\dot{\mathrm{e}}}_{5}+{\mathrm{e}}_{6}{\dot{\mathrm{e}}}_{6}{=\mathrm{e}}_{5}\left({\dot{\mathrm{P}}}_{\mathrm{fref}}-{\mathrm{v}}_{\mathrm{s}}(-\frac{{\mathrm{v}}_{\mathrm{qfn}}}{{\mathrm{L}}_{\mathrm{f}}}-\frac{{\mathrm{R}}_{\mathrm{f}}}{{\mathrm{L}}_{\mathrm{f}}}{\mathrm{I}}_{\mathrm{qf}}-{\upomega }_{\mathrm{s}}{\mathrm{I}}_{\mathrm{df}}+\frac{{\mathrm{v}}_{\mathrm{s}}}{{\mathrm{L}}_{\mathrm{f}}})\right)+{\mathrm{e}}_{6}\left({\dot{\mathrm{Q}}}_{\mathrm{fref}}+{\mathrm{v}}_{\mathrm{s}}.(-\frac{{\mathrm{v}}_{\mathrm{dfn}}}{{\mathrm{L}}_{\mathrm{f}}}- \frac{{\mathrm{R}}_{\mathrm{f}}}{{\mathrm{L}}_{\mathrm{f}}}{\mathrm{I}}_{\mathrm{df}}+{\upomega }_{\mathrm{s}}{\mathrm{I}}_{\mathrm{qf}})\right)$$

${\dot{V}}_{3}$ must be negative for the system to be stable. For this, we choose ${\dot{V}}_{3}$ in the form:

40 $${\dot{\mathrm{V}}}_{3}=-{\mathrm{k}}_{5}{\mathrm{e}}_{5}^{2}-{\mathrm{k}}_{6}{\mathrm{e}}_{6}^{2}\le 0$$

With:${\mathrm{k}}_{5}$, ${\mathrm{k}}_{6}$ are positive constants.

By making the equality between (39) and (40), we obtain:

41 $${\mathrm{e}}_{5}\left({\dot{\mathrm{P}}}_{\mathrm{fref}}-{\mathrm{v}}_{\mathrm{s}}\left(-\frac{{\mathrm{v}}_{\mathrm{qfn}}}{{\mathrm{L}}_{\mathrm{f}}}-\frac{{\mathrm{R}}_{\mathrm{f}}}{{\mathrm{L}}_{\mathrm{f}}}{\mathrm{I}}_{\mathrm{qf}}-{\upomega }_{\mathrm{s}}{\mathrm{I}}_{\mathrm{df}}+\frac{{\mathrm{v}}_{\mathrm{s}}}{{\mathrm{L}}_{\mathrm{f}}}\right)\right)+{\mathrm{e}}_{6}\left({\dot{\mathrm{Q}}}_{\mathrm{fref}}+{\mathrm{v}}_{\mathrm{s}}.\left(-\frac{{\mathrm{v}}_{\mathrm{dfn}}}{{\mathrm{L}}_{\mathrm{f}}}-\frac{{\mathrm{R}}_{\mathrm{f}}}{{\mathrm{L}}_{\mathrm{f}}}{\mathrm{I}}_{\mathrm{df}}+{\upomega }_{\mathrm{s}}{\mathrm{I}}_{\mathrm{qf}}\right)\right)=-{\mathrm{k}}_{5}{\mathrm{e}}_{5}^{2}-{\mathrm{k}}_{6}{\mathrm{e}}_{6}^{2}$$

Which give:

42 $$\left\{\begin{array}{c}{\mathrm{e}}_{5}\left({\dot{\mathrm{P}}}_{\mathrm{fref}}-{\mathrm{v}}_{\mathrm{s}}(-\frac{{\mathrm{v}}_{\mathrm{qfn}}}{{\mathrm{L}}_{\mathrm{f}}}-\frac{{\mathrm{R}}_{\mathrm{f}}}{{\mathrm{L}}_{\mathrm{f}}}{\mathrm{I}}_{\mathrm{qf}}-{\upomega }_{\mathrm{s}}{\mathrm{I}}_{\mathrm{df}}+\frac{{\mathrm{v}}_{\mathrm{s}}}{{\mathrm{L}}_{\mathrm{f}}})\right)=-{\mathrm{k}}_{5}{\mathrm{e}}_{5}^{2}\\ {\mathrm{e}}_{6}\left({\dot{\mathrm{Q}}}_{\mathrm{fref}}+{\mathrm{v}}_{\mathrm{s}}.(-\frac{{\mathrm{v}}_{\mathrm{dfn}}}{{\mathrm{L}}_{\mathrm{f}}}-\frac{{\mathrm{R}}_{\mathrm{f}}}{{\mathrm{L}}_{\mathrm{f}}}{\mathrm{I}}_{\mathrm{df}}+{\upomega }_{\mathrm{s}}{\mathrm{I}}_{\mathrm{qf}})\right)=-{\mathrm{k}}_{6}{\mathrm{e}}_{6}^{2}\end{array}\right..$$

The expression of the virtual control ${\mathrm{I}}_{\mathrm{qf}}$ and ${\mathrm{I}}_{\mathrm{df}}$ is defined by:

43 $$\left\{\begin{array}{c}{\mathrm{I}}_{\mathrm{qfref}}=-\frac{{\mathrm{L}}_{\mathrm{f}}}{{\mathrm{R}}_{\mathrm{f}}{.\mathrm{v}}_{\mathrm{s}}}\left({\dot{\mathrm{P}}}_{\mathrm{fref}}+{\mathrm{v}}_{\mathrm{s}}\left(\frac{{\mathrm{v}}_{\mathrm{qfn}}}{{\mathrm{L}}_{\mathrm{f}}}+{\upomega }_{\mathrm{s}}{\mathrm{I}}_{\mathrm{df}}-\frac{{\mathrm{v}}_{\mathrm{s}}}{{\mathrm{L}}_{\mathrm{f}}}\right)+{\mathrm{k}}_{5}{\mathrm{e}}_{5}\right)\\ {\mathrm{I}}_{\mathrm{dfref}}=\frac{{\mathrm{L}}_{\mathrm{f}}}{{\mathrm{R}}_{\mathrm{f}}{.\mathrm{v}}_{\mathrm{s}}}.\left({\dot{\mathrm{Q}}}_{\mathrm{fref}}+{\mathrm{v}}_{\mathrm{s}}.\left(-\frac{{\mathrm{v}}_{\mathrm{dfn}}}{{\mathrm{L}}_{\mathrm{f}}}+{\upomega }_{\mathrm{s}}{\mathrm{I}}_{\mathrm{qf}}\right)+{\mathrm{k}}_{6}{\mathrm{e}}_{6}\right)\end{array}\right..$$

This control will be the desired reference of the following system.

**Step 2**: Calculation of the filter voltages Vfdn and Vfqn

In this step, we will deduce the true control law Vfdn and Vfqn to achieve the design objectives for the overall system^41, 42^.

44 $$\left\{\begin{array}{c}{\mathrm{e}}_{7}={\mathrm{I}}_{\mathrm{dfref}}-{\mathrm{I}}_{\mathrm{df}}\\ {\mathrm{e}}_{8}={\mathrm{I}}_{\mathrm{qfref}}-{\mathrm{I}}_{\mathrm{qf}}\end{array}\right.$$

The derivatives of the errors are given by:

45 $$\left\{\begin{array}{c}{\dot{\mathrm{e}}}_{7}={\dot{\mathrm{I}}}_{\mathrm{dfref}}-{\dot{\mathrm{I}}}_{\mathrm{df}}\\ {\dot{\mathrm{e}}}_{8}={\dot{\mathrm{I}}}_{\mathrm{qfref}}-{\dot{\mathrm{I}}}_{\mathrm{qf}}\end{array}\right..$$

By replacing (43) in (45), we obtain the following equation:

46 $$\left\{\begin{array}{c}{\dot{\mathrm{e}}}_{7}={\dot{\mathrm{I}}}_{\mathrm{dfref}}+\frac{{\mathrm{v}}_{\mathrm{dfn}}}{{\mathrm{L}}_{\mathrm{f}}}+\frac{{\mathrm{R}}_{\mathrm{f}}}{{\mathrm{L}}_{\mathrm{f}}}{\mathrm{I}}_{\mathrm{df}}-{\upomega }_{\mathrm{s}}{\mathrm{I}}_{\mathrm{qf}}\\ {\dot{\mathrm{e}}}_{8}={\dot{\mathrm{I}}}_{\mathrm{qfref}}+\frac{{\mathrm{v}}_{\mathrm{qfn}}}{{\mathrm{L}}_{\mathrm{f}}}+\frac{{\mathrm{R}}_{\mathrm{f}}}{{\mathrm{L}}_{\mathrm{f}}}{\mathrm{I}}_{\mathrm{qf}}+{\upomega }_{\mathrm{s}}{\mathrm{I}}_{\mathrm{df}}-\frac{{\mathrm{v}}_{\mathrm{s}}}{{\mathrm{L}}_{\mathrm{f}}}\end{array}\right..$$

We first choose the candidate function of “LYAPUNOV” associated with the errors of the filter currents in the following quadratic form:

47 $${\mathrm{V}}_{4}=\frac{1}{2}{\mathrm{e}}_{7}^{2}+\frac{1}{2}{\mathrm{e}}_{8}^{2}$$

Its derivative is given by:

48 $${\dot{\mathrm{V}}}_{4}={\mathrm{e}}_{7}{\dot{\mathrm{e}}}_{7}+{\mathrm{e}}_{8}{\dot{\mathrm{e}}}_{8}{=\mathrm{e}}_{7}\left({\dot{\mathrm{I}}}_{\mathrm{dfref}}+\frac{{\mathrm{v}}_{\mathrm{dfn}}}{{\mathrm{L}}_{\mathrm{f}}}+\frac{{\mathrm{R}}_{\mathrm{f}}}{{\mathrm{L}}_{\mathrm{f}}}{\mathrm{I}}_{\mathrm{df}}-{\upomega }_{\mathrm{s}}{\mathrm{I}}_{\mathrm{qf}}\right)+{\mathrm{e}}_{8}\left({\dot{\mathrm{I}}}_{\mathrm{qfref}}+\frac{{\mathrm{v}}_{\mathrm{qfn}}}{{\mathrm{L}}_{\mathrm{f}}}+\frac{{\mathrm{R}}_{\mathrm{f}}}{{\mathrm{L}}_{\mathrm{f}}}{\mathrm{I}}_{\mathrm{qf}}+{\upomega }_{\mathrm{s}}{\mathrm{I}}_{\mathrm{df}}-\frac{{\mathrm{v}}_{\mathrm{s}}}{{\mathrm{L}}_{\mathrm{f}}}\right)$$

According to LYAPUNOV, it is necessary to choose a negative function ${\dot{\mathrm{V}}}_{4}$, to ensure the stability of the system. For this, we choose ${\dot{\mathrm{V}}}_{4}$ in the following form:

49 $${\dot{\mathrm{V}}}_{4}=-{\mathrm{k}}_{7}{\mathrm{e}}_{7}^{2}-{\mathrm{k}}_{8}{\mathrm{e}}_{8}^{2}\le 0$$

With: ${\mathrm{k}}_{7}$,${\mathrm{k}}_{8}$ are positive constants.

Equations (48) and (49), we get:

50 $${\mathrm{e}}_{7}\left({\dot{\mathrm{I}}}_{\mathrm{dfref}}+\frac{{\mathrm{v}}_{\mathrm{dfn}}}{{\mathrm{L}}_{\mathrm{f}}}+\frac{{\mathrm{R}}_{\mathrm{f}}}{{\mathrm{L}}_{\mathrm{f}}}{\mathrm{I}}_{\mathrm{df}}-{\upomega }_{\mathrm{s}}{\mathrm{I}}_{\mathrm{qf}}\right)+{\mathrm{e}}_{8}\left({\dot{\mathrm{I}}}_{\mathrm{qfref}}+\frac{{\mathrm{v}}_{\mathrm{qfn}}}{{\mathrm{L}}_{\mathrm{f}}}+\frac{{\mathrm{R}}_{\mathrm{f}}}{{\mathrm{L}}_{\mathrm{f}}}{\mathrm{I}}_{\mathrm{qf}}+{\upomega }_{\mathrm{s}}{\mathrm{I}}_{\mathrm{df}}-\frac{{\mathrm{v}}_{\mathrm{s}}}{{\mathrm{L}}_{\mathrm{f}}}\right)=-{\mathrm{k}}_{7}{\mathrm{e}}_{7}^{2}-{\mathrm{k}}_{8}{\mathrm{e}}_{8}^{2}$$

Which give:

51 $$\left\{\begin{array}{c}{\mathrm{e}}_{7}\left({\dot{\mathrm{I}}}_{\mathrm{dfref}}+\frac{{\mathrm{v}}_{\mathrm{dfn}}}{{\mathrm{L}}_{\mathrm{f}}}+\frac{{\mathrm{R}}_{\mathrm{f}}}{{\mathrm{L}}_{\mathrm{f}}}{\mathrm{I}}_{\mathrm{df}}-{\upomega }_{\mathrm{s}}{\mathrm{I}}_{\mathrm{qf}}\right)=-{\mathrm{k}}_{7}{\mathrm{e}}_{7}^{2}\\ {\mathrm{e}}_{8}\left({\dot{\mathrm{I}}}_{\mathrm{qfref}}+\frac{{\mathrm{v}}_{\mathrm{qfn}}}{{\mathrm{L}}_{\mathrm{f}}}+\frac{{\mathrm{R}}_{\mathrm{f}}}{{\mathrm{L}}_{\mathrm{f}}}{\mathrm{I}}_{\mathrm{qf}}+{\upomega }_{\mathrm{s}}{\mathrm{I}}_{\mathrm{df}}-\frac{{\mathrm{v}}_{\mathrm{s}}}{{\mathrm{L}}_{\mathrm{f}}}\right)=-{\mathrm{k}}_{8}{\mathrm{e}}_{8}^{2}\end{array}\right.$$

The expression of the real global control ${\mathrm{V}}_{\mathrm{qfn}}$ and ${\mathrm{V}}_{\mathrm{dfn}}$ defined by:

52 $$\left\{\begin{array}{c}{\mathrm{v}}_{\mathrm{dfn}}=-{\mathrm{L}}_{\mathrm{f}}({\mathrm{k}}_{7}{\mathrm{e}}_{7}+{\dot{\mathrm{I}}}_{\mathrm{dfref}}+\frac{{\mathrm{R}}_{\mathrm{f}}}{{\mathrm{L}}_{\mathrm{f}}}{\mathrm{I}}_{\mathrm{df}}+{\upomega }_{\mathrm{s}}{\mathrm{I}}_{\mathrm{qf}})\\ {\mathrm{v}}_{\mathrm{qfn}}=-{\mathrm{L}}_{\mathrm{f}}({\mathrm{k}}_{8}{\mathrm{e}}_{8}+{\dot{\mathrm{I}}}_{\mathrm{qfref}}+\frac{{\mathrm{R}}_{\mathrm{f}}}{{\mathrm{L}}_{\mathrm{f}}}{\mathrm{I}}_{\mathrm{qf}}+{\upomega }_{\mathrm{s}}{\mathrm{I}}_{\mathrm{df}}-\frac{{\mathrm{v}}_{\mathrm{s}}}{{\mathrm{L}}_{\mathrm{f}}})\end{array}\right.$$

### Generation of the global control by the sliding-Backstepping mode control

The Sliding–Backstepping Mode controller is composed of two parts: the first “${\mathrm{u}}_{\mathrm{eq}}$” generated by the sliding mode control and the second “${\mathrm{u}}_{\mathrm{n}}$” generated by the Backstepping control [^43–46^].

53 $${\mathrm{u}}_{\mathrm{MG}-\mathrm{BS}}={\mathrm{u}}_{\mathrm{eq}-\mathrm{MG}}+{\mathrm{u}}_{\mathrm{n}-\mathrm{BS}}$$

We will apply the same sliding mode control structure studied in the previous part to generate the equivalent control ${\mathrm{u}}_{\mathrm{eq}-\mathrm{MG}}$, and in the second part ${\mathrm{u}}_{\mathrm{n}-\mathrm{BS}}$ we will use the Backstepping control to have the stabilizing control Un .

#### Application of the hybrid control to the RSC

By applying the Hybrid Sliding-Backstepping Mode control to the Rotor Side Converter, the global equation of the ${\mathrm{V}}_{\mathrm{rd}}$ and ${\mathrm{V}}_{\mathrm{rq}}$ control takes the following forms^47, 48^:

The voltage ${\mathrm{V}}_{\mathrm{rd}}$ having the equation:

54 $${\mathrm{V}}_{\mathrm{rd}}=-\frac{{\mathrm{L}}_{\mathrm{r}}.{\mathrm{L}}_{\mathrm{s}}.\upsigma }{\mathrm{M}.\mathrm{V}}{.{\dot{\mathrm{Q}}}_{\mathrm{sref}}+{\mathrm{R}}_{\mathrm{r}}.{\mathrm{I}}_{\mathrm{rd}}-{{\mathrm{L}}_{\mathrm{r}}.\upsigma .\upomega }_{\mathrm{r}}.{\mathrm{I}}_{\mathrm{rq}}+\mathrm{L}}_{\mathrm{r}}.\upsigma \left[{\mathrm{k}}_{4}{\mathrm{e}}_{4}+{\dot{\mathrm{I}}}_{\mathrm{rdref}}+\frac{1}{{\mathrm{L}}_{\mathrm{r}}.\upsigma }\left({\mathrm{R}}_{\mathrm{r}}.{\mathrm{I}}_{\mathrm{rd}}-{\mathrm{L}}_{\mathrm{r}}.\upsigma .{\upomega }_{\mathrm{r}}.{\mathrm{I}}_{\mathrm{rq}}\right)\right]$$

The given voltage ${\mathrm{V}}_{\mathrm{rq}}$:

55 $${\mathrm{V}}_{\mathrm{rq}}=-\frac{{\mathrm{L}}_{\mathrm{r}}.{\mathrm{L}}_{\mathrm{s}}.\upsigma }{\mathrm{M}.\mathrm{V}}.{\dot{\mathrm{P}}}_{\mathrm{sref}}+{\mathrm{R}}_{\mathrm{r}}.{\mathrm{I}}_{\mathrm{rq}}+{{\mathrm{L}}_{\mathrm{r}}.\upsigma .\upomega }_{\mathrm{r}}.{\mathrm{I}}_{\mathrm{rd}}+{{\upomega }_{\mathrm{r}}.\mathrm{M}.\frac{\mathrm{M}.{\mathrm{V}}_{\mathrm{s}}}{{\mathrm{L}}_{\mathrm{s}}.{\upomega }_{\mathrm{s}}}+\mathrm{L}}_{\mathrm{r}}.\upsigma [{\mathrm{k}}_{3}{\mathrm{e}}_{3}+{\dot{\mathrm{I}}}_{\mathrm{rqref}}+\frac{1}{{\mathrm{L}}_{\mathrm{r}}.\upsigma }\left({\mathrm{R}}_{\mathrm{r}}.{\mathrm{I}}_{\mathrm{rq}}+{{\mathrm{L}}_{\mathrm{r}}.\upsigma .\upomega }_{\mathrm{r}}.{\mathrm{I}}_{\mathrm{rd}}+\mathrm{g}.\frac{\mathrm{M}.{\mathrm{V}}_{\mathrm{s}}}{{\mathrm{L}}_{\mathrm{s}}}\right)]$$

According to the voltage equations, we elaborate the control block by the Sliding-Backstepping Mode applied to the RSC illustrated by the following Fig. 3.

#### Application of the hybrid control to the GSC

By applying the Hybrid Sliding-Backstepping Mode control to the Grid Side Converter, the expression of the Global control ${\mathrm{v}}_{\mathrm{df}}$ and ${\mathrm{v}}_{\mathrm{qf}}$ is given by:

56 $$\left\{\begin{array}{c}{\mathrm{v}}_{\mathrm{df}}=\frac{{\mathrm{L}}_{\mathrm{f}}}{{\mathrm{V}}_{\mathrm{s}}}\dot{{\mathrm{Q}}_{\mathrm{sref}}}-{\mathrm{R}}_{\mathrm{f}}{\mathrm{I}}_{\mathrm{df}}+{\mathrm{L}}_{\mathrm{f}}{\mathrm{I}}_{\mathrm{qf}}{\upomega }_{\mathrm{s}}-{\mathrm{L}}_{\mathrm{f}}({\mathrm{k}}_{5}{\mathrm{e}}_{5}+{\dot{\mathrm{I}}}_{\mathrm{dfref}}+\frac{{\mathrm{R}}_{\mathrm{f}}}{{\mathrm{L}}_{\mathrm{f}}}{\mathrm{I}}_{\mathrm{df}}+{\upomega }_{\mathrm{s}}{\mathrm{I}}_{\mathrm{qf}})\\ {\mathrm{v}}_{\mathrm{qf}}=\frac{{\mathrm{L}}_{\mathrm{f}}}{{\mathrm{V}}_{\mathrm{s}}}\dot{{\mathrm{P}}_{\mathrm{sref}}}-{\mathrm{R}}_{\mathrm{f}}{\mathrm{I}}_{\mathrm{qf}}-{\mathrm{L}}_{\mathrm{f}}{\mathrm{I}}_{\mathrm{df}}{\upomega }_{\mathrm{s}}+{\mathrm{v}}_{\mathrm{s}}-{\mathrm{L}}_{\mathrm{f}}({\mathrm{k}}_{6}{\mathrm{e}}_{6}+{\dot{\mathrm{I}}}_{\mathrm{qfref}}+\frac{{\mathrm{R}}_{\mathrm{f}}}{{\mathrm{L}}_{\mathrm{f}}}{\mathrm{I}}_{\mathrm{qf}}+{\upomega }_{\mathrm{s}}{\mathrm{I}}_{\mathrm{df}}-\frac{{\mathrm{v}}_{\mathrm{s}}}{{\mathrm{L}}_{\mathrm{f}}})\end{array}\right.$$

The control block by the Sliding-Backstepping Mode control applied to the GSC is given by the following Fig. 4.

## Simulation results

To illustrate the performance of the Sliding-Backstepping Mode control applied to a 10 kW DFIG connected to a 400V / 50Hz Grid dedicated to a wind system, we will apply the same tests carried out previously.

### Pursuit tests

#### Step speed response

#### Variable speed response

**Figure 5:**
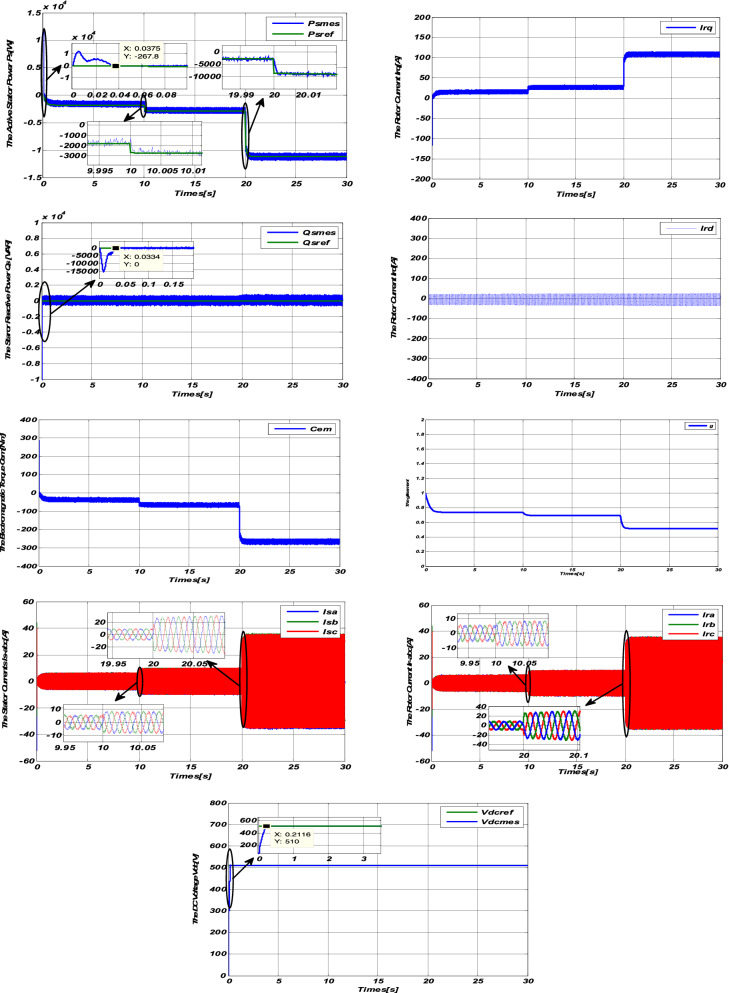
Results of the Sliding-Backstepping Mode control at step wind speed”.

**Figure 6:**
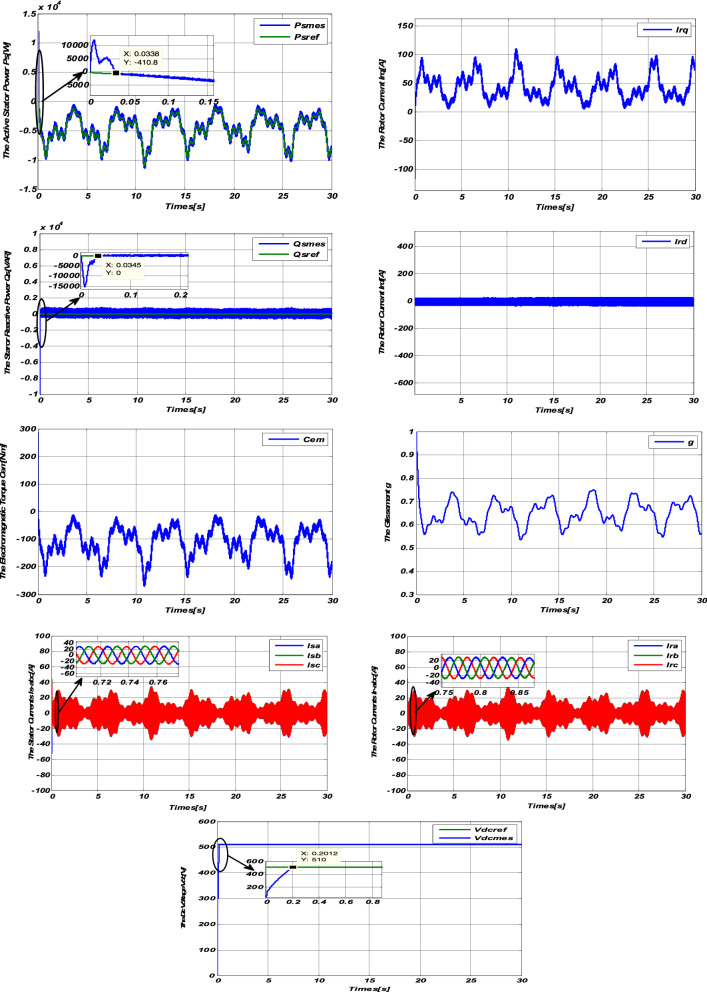
Results of the Sliding-Backstepping Mode variable speed control.

From these results (Figs. 5 and 6), we can conclude that the powers generated by the DFIG perfectly follow their references with better decoupling and a low response time lower than that of the PI regulator and the sliding mode regulator.

We can also notice that the electromagnetic torque depends on the active power, this is translated by its form identical to that of the active power.

The rotor current Irq depends on the Active power Ps and the rotor current Irq depends on the Reactive power Qs.

The positive sign of the slip g then indicates that the machine is operating in hypo-synchronous mode.

The GRID receives good quality energy because, according to the results obtained, the currents of the ${\mathrm{I}}_{\mathrm{Sabc}}$ stator are sinusoidal, of better quality than those obtained by the control of the sliding mode.

Figure 6 shows that the DC bus voltage perfectly follows its reference value of 510 V with almost zero error and a slower response time than the sliding mode control.

Table 1 summarizes the response time of the controller by Sliding-Backstepping Mode for the active and reactive powers as well as the DC-bus voltage.

**Table 1:** summary of Backstepping controller response time.

MG-BS controller	tr
Active Power Ps	0.0338
Reactive Power Qs	0.0345
DC-Bus	0.2012

One can also see that the results obtained from the Sliding-Backstepping Mode control are less wavy than the control results by sliding mode, which implies the robustness of the proposed control.

### Robustness tests

**Figure 7:**
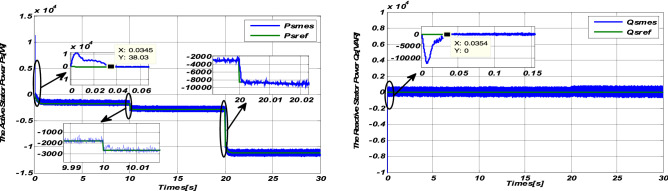
Robustness test with variation of resistances Rr and Rs for step wind speed.

**Figure 8:**
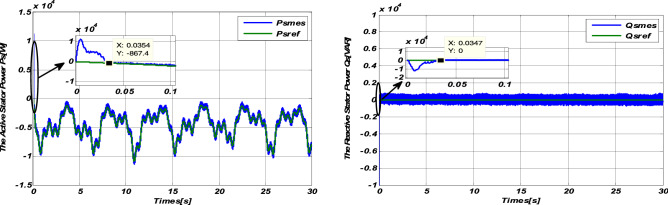
Robustness test with the variation of resistances Rr and Rs for variable wind speed.

The same tests were carried out to test the robustness of the DFIG Sliding-Backstepping Mode control: Rr and Rs increase by 50% of their nominal values. The results obtained are represented through Figs. 7 and 8:

**Figure 9:**
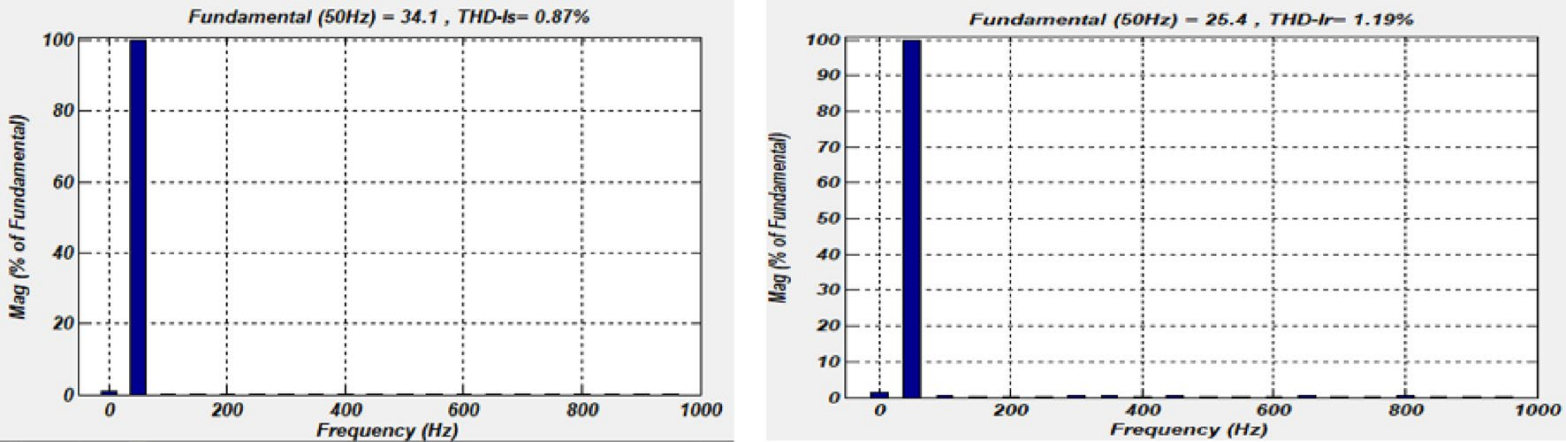
Spectral analysis of the phase current Isa and Ira using the Sliding-Backstepping Mode control.

#### Step wind speed response

#### Response to variable wind speed

The simulation results (Figs. 7 and 8) show the robustness of the Sliding-Backstepping Mode control compared to the parametric variations of the DFIG. This is justified by the good monitoring of the power set points, with almost the same response time at startup. Furthermore, the active and reactive powers are always kept decoupled.

### Spectral analysis by the Sliding-Backstepping Mode control

The results of Fig. 9 show the efficiency of the proposed Hybrid control concerning the reduction of the CHATTERING phenomenon, this is justified by the decrease in the rate of harmonic distortion THD which equals 1.19% for the current rotor of one phase and 0.87% for the stator current Isa.

These results allow us to conclude that the Sliding-Backstepping controller is the most efficient concerning reducing the CHATTERING phenomenon and the most robust concerning the parametric variations of the machine (Table 2).

**Table 2:** Control's comparison.

Publication paper	Technical methods	Response time(s)	Error (%)	THD (%)
^5^	Fuzzy field-oriented control	1	–	1.54
^8^	Direct Control	–	–	1.60
^11^	Model predictive speed control	0.0896	–	–
^17^	Double Integral Sliding Mode control	0.003	0.006	1.39
^49^	Proposed Sliding Mode control	0.015	0.15	1.25
Proposed technique	B-SMC	0.03	0.09	0.87

## Conclusions

Initially, the WECS and the DFIG models were developed, then and given keeping the robustness of the sliding mode control of a wind energy conversion system based on DFIG and eliminating the phenomenon of CHATTERING without degrading the performance of the system, hybridization between the sliding mode control and the Backstepping control was evaluated.

This hybridization technique has given rise to a new robust regulator called drag Backstepping mode. The control is then reviewed to ensure that the WECS extracts the maximum amount of electrical energy towards parametric variations. Next, a simulation was run in Matlab/Simulink environments to determine the performance and durability of the proposed control.

The results obtained showed very satisfactory and significant performance of good regulation. In addition, the pursuit, regulation, and robustness behaviours are significantly better than those observed for the other strategies studied.

As a perspective, the proposed method will be implemented in an embedded board to build a prototype that can be commercialized, and also develop a new control method by applying the proposed method to the finite/fixed time SMC method.

## Data Availability

Data available on request from the authors.

## List of symbols

Ω_t_: Turbine speed
*R*_*s*_, *R*_*r*_: Stator and rotor resistance
*R*_*f*_, *L*_*f*_: Filter resistance and inductance
*L*_*r*_, *L*_*s*_: Stator and rotor inductance
*P*_*s*_, *P*_*r*_, *P*_g_: Stator and rotor and grid active power
*Q*_*s*_, *Q*_*r*_, Q_g_: Stator and rotor and grid reactive power
*T*_*em *_*,C*_*em*_: Electromagnetic torque
*V*_*r(a,b,c) *_, *V*_*s(A,B,C) *_: Rotor and stator voltages
*i*_*r(a,b,c) *_, *i*_*s(A,B,C)*_: currents of stator and rotor
(*v*_*sd*_* ,v*_*sq*_), (*i*_*sd*_* , i*_*sq*_): d/q stator voltages and currents
(*v*_*td*_, *v*_*tq*_), (*i*_*td*_ ,*i*_*tq*_): Voltages and currents at the RL filter
